# Exploration and exploitation of homologous series of bis(acrylamido)alkanes containing pyridyl and phenyl groups: β-sheet *versus* two-dimensional layers in solid-state photochemical [2 + 2] reactions

**DOI:** 10.1107/S2052252515009987

**Published:** 2015-07-31

**Authors:** Mousumi Garai, Kumar Biradha

**Affiliations:** a Department of Chemistry, Indian Institute of Technology Kharagpur, Kharagpur, West Bengal 721302, India

**Keywords:** homologous series, hydrogen bonds, solid-state [2 + 2] reaction, organic polymer

## Abstract

The homologous series of phenyl and pyridyl substituted bis(acrylamido)alkanes have been synthesized with the aim of systematic analysis of their crystal structures and their solid-state [2 + 2] reactivities. The changes in the crystal structures with respect to a small change in the molecular structure, that is by varying alkyl spacers between acrylamides and/or by varying the end groups (phenyl, 2-pyridyl, 3-pyridyl, 4-pyridyl) on the C-terminal of the amide, were analyzed in terms of hydrogen-bonding interference (N—H⋯N_py_ *versus* N—H⋯O=C) and network geometries. In this series, a greater tendency towards the formation of N—H⋯O hydrogen bonds (β-sheets and two-dimensional networks) over N—H⋯N hydrogen bonds was observed. Among all the structures seven structures were found to have the required alignments of double bonds for the [2 + 2] reaction such that the formations of single dimer, double dimer and polymer are facilitated. However, only four structures were found to exhibit such a solid-state [2 + 2] reaction to form a single dimer and polymers. The two-dimensional hydrogen-bonding layer *via* N—H⋯O hydrogen bonds was found to promote solid-state [2 + 2] photo-polymerization in a single-crystal-to-single-crystal manner. Such two-dimensional layers were encountered only when the spacer between acryl amide moieties is butyl. Only four out of the 16 derivatives were found to form hydrates, two each from 2-pyridyl and 4-pyridyl derivatives. The water molecules in these structures govern the hydrogen-bonding networks by the formation of an octameric water cluster and one-dimensional zigzag water chains. The trends in the melting points and densities were also analyzed.

## Abstract 2

Sixteen derivatives of the homologous series of bis(acrylamido)alkanes have been synthesized with the aim of systematic analysis of their crystal structures and their solid-state [2 + 2] reactivities. Seven out of the 16 found to have reactive alignments for solid-state [2 + 2] reactions yield dimers and polymers.

## 1. Introduction   

The systematic exploration of solid-state ensembles of organic molecules containing functional groups which possess similar interactive capabilities albeit minor variations laid the foundation for the gigantic rise of the field of crystal engineering (Schmidt, 1971 ▸; Desiraju, 1989 ▸; Lehn, 1995 ▸; Whitesides *et al.*, 1995 ▸; Aakeröy *et al.*, 2001 ▸; Moulton & Zaworotko, 2001 ▸; Zaworotko, 2001 ▸; Desiraju, 2002 ▸; Biradha, 2003 ▸; Vangala *et al.*, 2005 ▸; Rajput *et al.*, 2007 ▸ *a* ▸; Mukherjee & Biradha, 2011*b* ▸). The comparison between various halogen derivatives, either by changing the halogen atoms or by changing their position on the aromatic ring is one of the finest examples of such systematic explorations (Bent, 1968 ▸; Legon, 1999 ▸; Metrangolo & Resnati, 2001 ▸; Metrangolo *et al.*, 2005 ▸, 2008 ▸; Marras *et al.*, 2006 ▸; Shirman *et al.*, 2008 ▸; Samai & Biradha, 2009 ▸). On the other hand, the similarities or anomalies in the properties of homologues series of *n*-alkanes and α,ω-substituted dithiols, diols, diamines and diacids are well understood from their crystal structures (Hicks & Nodwell, 2000 ▸; Xu *et al.*, 2002 ▸; Vishweshwar *et al.*, 2003 ▸; Gibson *et al.*, 2003 ▸; Lee *et al.*, 2007 ▸). Apart from these very few homologous series, in particular molecules containing more than one functional group that are capable of hydrogen bonding, are characterized crystallographically. The increase in functional groups and flexibility increases the complexity and decreases the probability of having isostructurality in the series. Further, the isostructurality of homologous series is not an obvious fact as the process of crystallization depends on several factors such as aggregation, size and solvation apart from the structure of a molecule.

**Figure 1:**
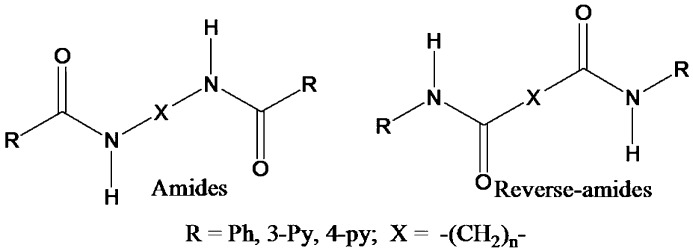
Amides and reverse amides

In these lines we have previously reported our studies on homologous series of *N*,*N*′-bis(pyridinecarboxamido)alkanes (amides) (Sarkar & Biradha, 2006 ▸) and *N*,*N*′-bis(pyridyl)alkanediamides (reverse amides) (Rajput *et al.*, 2007 ▸ ▸ *b* ▸) in which amide moieties are separated by alkyl (—(CH_2_)_*n*_—) spacers (Fig. 1 ▸). From these studies it was evident that both series exhibit an odd–even effect on the nature of the hydrogen bond and their network features (Mukherjee & Biradha, 2011*a* ▸). As these classes of compounds contain two each of amide and pyridine moieties, the molecules were assembled through either N—H⋯O or N—H⋯N or both the interactions. The β-sheets or (4,4)-networks, *via* N—H⋯O or N—H⋯N hydrogen bonds, are the two most common motifs displayed by the derivatives containing an even number of —CH_2_— groups. The amide molecules were found to assemble mostly *via* amide-to-amide recognitions in amides, while pyridine interferences to the amide-to-amide hydrogen bond was found to be more prominent in the case of the reverse amide series, *i.e.* the N—H⋯N interaction is preferred over the N—H⋯O hydrogen bond. In contrast to the formation of the β-sheets or (4,4)-networks by the even ones, the formation of three-dimensional structures were found to be more frequent in the case of odd ones.

**Figure 2:**
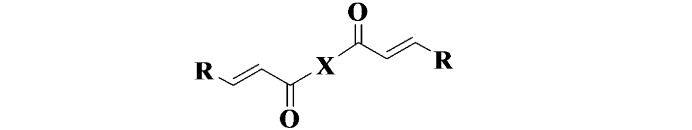
**1**: *R* = phenyl; **2**: *R* = 2-pyridyl; **3**: *R* = 3-pyridyl; **4**: *R* = 4-pyridyl, **a**: *X* = —HN—NH—, **b**: *X* = —HN—(CH_2_)_2_—NH, **c**: *X* = —HN—(CH_2_)_4_—NH—; **d**: *X* = —HN—(CH_2_)_6_—NH—.

**Figure 3:**
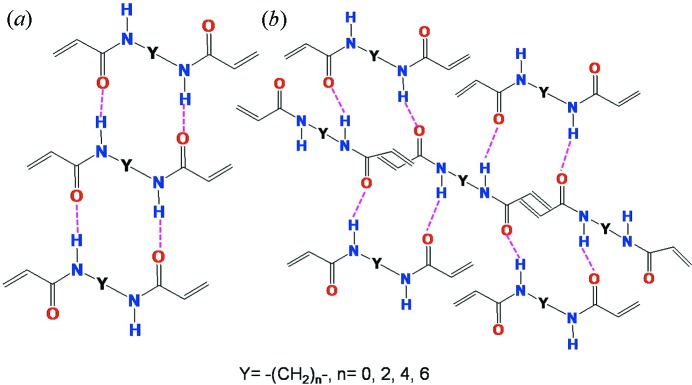
Representation of (*a*) β-sheet and (*b*) two-dimensional layers.

From these studies it was understood that the hydrogen bonded (4,4)-networks can promote solid-state [2 + 2] reactions (Garai *et al.*, 2013 ▸) to form polymers containing cyclobutanes and amides in the chain with the phenyl/pyridyl groups as dangling attachments, provided the bis-amide molecules contain double bonds at the terminals. In order to test this hypothesis several derivatives of **1**–**4** were prepared by changing the spacers between the amide functionalities (Fig. 2 ▸). The crystal structures were analyzed in comparison with those of homologues series of amides and reverse amides. Further, the solid-state [2 + 2] reactions were explored wherever possible. It was shown by us earlier that the crystal structures of molecules **1*c*** and **3*c*** containing the butane spacer possess such unique features to form the hydrogen bonded (4,4)-layer which promotes the polymerization reaction as anticipated in a single-crystal-to-single-crystal (SCSC) manner to yield crystalline organic polymers (Garai *et al.*, 2013 ▸). Such polymerization reactions are very rare and the only example in the literature is 2,5-distyrylpyrazine reported by Hasegawa *et al.* (Hasegawa, 1983 ▸; Hasegawa *et al.*, 1986 ▸). In this manuscript the following points will be addressed by analyzing homologous series of crystal structures of **1**–**4**: (1) competition between the O atom of the amide and the N atom of pyridine to form hydrogen bonds with the amide N—H group; (2) similarities or differences with previously published bis-amide analogues; (3) formation of the two-dimensional layer *versus* the β-sheet hydrogen-bond networks (Fig. 3 ▸); (4) propensity for the formation of hydrates; (5) trends in their melting points; (6) alignment of double bonds for solid-state [2 + 2] reactions; (7) exploration of their reactivities and characterization of their products wherever possible.

## 2. Experimental   

FTIR spectra were recorded with a Perkin–Elmer Instrument Spectrum Rx Serial No. 73713. Powder XRD patterns were recorded with a Bruker AXS-D8-ADVANCE diffractometer (Cu target). ^1^H NMR (200/600 MHz) spectra were recorded on a Bruker AC 200/600 MHz spectrometer. The MALDI-TOF experiment was carried out using a Bruker ultrafleXtreme MALDI TOF/TOF mass spectrometer.

### 2.1. Synthesis of **1*a***   

In a round-bottom flask cinnamic acid (1.21 g, 0.0081 mol) and pyridine (15 ml) were taken and hydrazine hydrate (0.194 ml, 0.004 mol) was added to the reaction mixture and stirred for ∼ 15–20 min. After that triphenyl phosphite (2.25 ml, 0.0086 mol) was added to it and the reaction mixture was refluxed for 7–8 h.

After cooling to room temperature, the pyridine was distilled out to reduce the volume up to 5 ml. For the work up process, EtOH was added and then the solid product was filtered and washed with EtOH. The white solid product was recrystallized from methanol–DMF.

**1*a***. Yield: 78%; m.p.: 280°C. Anal.: calc. for C_18_H_16_N_2_O_2_: C 74.0, H 5.5, N 9.6; found: C 69.49, H 4.79, N 8.59%.

Compounds **1*b***–**1*d***, **2*a***–**2*d*** and **3*a***–**3*d*** were prepared by following the above procedure, but by using corresponding diamine and cinnamic acid, (*E*)-3-(pyridin-2-yl)-acrylic acid and (*E*)-3-(pyridin-3-yl)-acrylic acid, respectively.

**1*b***. Yield: 84%; m.p.: 250°C. Anal.: calc. for C_20_H_18_N_2_O_2_: C 75.4, H 5.7, N 8.8; found: C 64.67, H 4.76, N 7.68%.

**1*c***. Yield: 72%; m.p.: 268°C. Anal.: calc. for C_22_H_24_N_2_O_2_: C 75.83, H 6.94, N 8.04; found: C 75.20, H 6.72, N 7.84%.

**2*a***. Yield: 56%; m.p.: 248°C. Anal.: calc. for C_16_H_14_N_4_O_2_: C 68.6, H 7.5, N 20.0; found: C 68.2, H 6.82, N 19.8%.

**2*b***. Yield: 42%; m.p.: 210°C. Anal.: calc. for C_18_H_18_N_4_O_6_: C 56.0, H 4.7, N 14.5; found: C 55.2, H 4.52, N 13.8%.

**2*d***. Yield: 60%; m.p.: 182°C. Anal.: calc. for C_22_H_26_N_4_O_4_: C 64.4, H 6.4, N 13.7; found: C 63.9, H 5.82, N 12.8%.

**3*a***. Yield: 57.2%; m.p. 272°C. Anal.: calc. for C_16_H_14_N_4_O_2_: C 65.68, H 4.42, N 18.76; found: C 65.30, H 4.79, N 19.04%.

**3*b***. Yield: 78%; m.p.: 235°C. Anal.: calc. for C_18_H_18_N_4_O_2_: C 67.1, H 5.6, N 17.4; found: C 66.43, H 5.62, N 16.52%.

**3*c***. Yield: 60%; m.p.: 249°C. Anal.: calc. for C_20_H_22_N_4_O_2_: C 68.55, H 6.33, N 15.99; found: C 67.74, H 6.63, N 14.96%.

### 2.2. Synthesis of **4*a***   

In a round-bottom flask (*E*)-3-(pyridine-4-yl) acrylic acid (0.596 g, 0.000399 mol), pentafluorophenol (0.8 g, 0.00434 mol) and DCC (0.908 g, 0.0044 mol) were taken in 20 ml of dry THF solvent and stirred over 24 h at room temperature, then the solvent was distilled out to collect the solid product. The solid product was recrystallized from pet-ether. The recrystallized ester (1 g, 0.00313 mol) and hydrazine hydrate (0.077 ml, 0.00109 mol) were taken in dry DMF solvent and stirred at room temperature for 24 h. Then the solid product was filtered and recrystallized with MeOH. Yield: 62.8%; m.p. 279°C. Anal.: calc. for C_16_H_14_N_4_O_2_: C 65.30, H 4.79, N 19.04; found: C 64.94, H 4.53, N 18.76%.

Similar procedures were followed for the synthesis of **4*b***–**4*d*** by using the corresponding diamines and pentafluoroester of (*E*)-3-(pyridine-4-yl) acrylic acid. In these cases, dry THF was used as the solvent.

**4*b***. Yield: 58%; m.p.: 275°C. Anal.: calc. for C_18_H_18_N_4_O_2_: C 67.1, H 5.6, N 17.4; found: C 66.9, H 5.49, N 16.69%.

**4*c***. Yield: 66%; m.p.: 235–238°C. Anal.: calc. for C_20_H_26_N_4_O_4_: C 62.2, H 6.8, N 14.5; found: C 61.49, H 5.59, N 14.19%.

**4*d***. Yield: 58%; m.p.: 175°C. Anal.: calc. for C_22_H_30_N_4_O_4_: C 63.7, H 7.3, N 13.5; found: C 62.79, H 6.49, N 12.46%.

### 2.3. Crystallographic data and refinement details   

All the single-crystal data were collected on a Bruker APEX-II CCD X-ray diffractometer that uses graphite monochromated Mo *K*α radiation (λ = 0.71073 Å) at room temperature (293 K) by the hemisphere method. The structures were solved by direct methods and refined by least-squares methods on *F* ^2^ using *SHELX*97 (Sheldrick, 2015 ▸). Non-hydrogen atoms were refined anisotropically and hydrogen atoms were fixed at calculated positions and refined using a riding model.

## 3. Results and discussion   

The compounds **1*a***–**1*d***, **2*a***–**2*d*** and **3*a***–**3*d*** are synthesized by refluxing the corresponding diamines with cinnamic acid, (*E*)-3-(pyridin-2-yl)-acrylic acid and (*E*)-3-(pyridin-3-yl)-acrylic acid, respectively, in the presence of triphenyl phosphate in pyridine. The same procedure was found to be incompatible for the syntheses of derivatives of **4**. Therefore, compounds **4*a***–**4*d*** are synthesized by reacting the pentafluoroester of (*E*)-3-(pyridin-4-yl)-acrylic acid with corresponding diamines. Single crystals suitable for X-ray diffraction were obtained by the slow evaporation technique from methanol, ethanol or methanol–DMF, methanol–THF solutions of the corresponding compounds. Despite several trails the single crystals suitable for X-ray diffraction analyses could not be obtained for **2*c*** and **3*d***. The comparison of X-ray powder diffraction (XRPD) patterns with the related analogues hinted at their probable crystal structures. The crystal structures of all the derivatives have been analyzed in terms of hydrogen-bonding networks and comparisons were made with respect to the related derivatives of amides and reverse amides. Further, these studies revealed that the series of these 16 structures can be categorized as four types: (1) β-sheet *via* N—H⋯O hydrogen bonds; (2) β-sheet *via* N—H⋯N hydrogen bonds; (3) two-dimensional layer *via* N—H⋯O hydrogen bonds; (4) two-dimensional layer *via* N—H⋯N hydrogen bonds. In the following sections the results will be described based on the spacers as listed in Fig. 2 ▸. The photochemical [2 + 2] reactions were carried out on **1*c***, **2*b***, **2*c***, **3*a***, **3*c***, **4*a*** and **4*c*** as the structures indicated the possibility of such reactions. The pertinent crystallographic details are given in Table 1 ▸ and the hydrogen-bonding parameters are given in Table 2 ▸.

### 3.1. β-sheet and two-dimensional layers with the —HN—NH— spacer   

**Figure 4:**
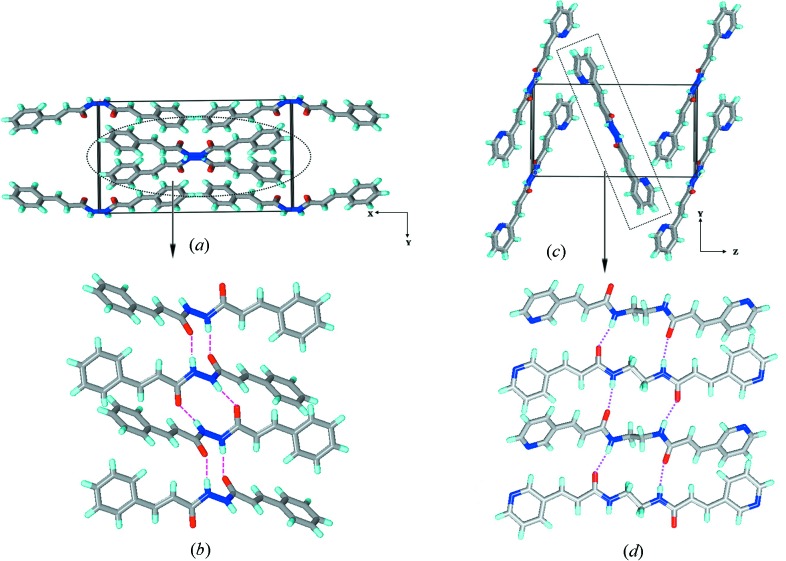
Illustrations for the crystal structures of **1*a*** and **2*a***: packing diagrams for (*a*) **1*a*** and (*c*) **2*a***; β-sheets in (*b*) **1*a*** and (*d*) **2*a***. Notice the difference in alignment of molecules within the β-sheet between **1*a*** and **2*a***.

Molecules **1*a***, **2*a***, **3*a*** and **4*a*** all crystallize in monoclinic space groups *C*2/*c*, *P*2_1_/*n*, *P*2_1_/*c* and *P*2_1_/*n*, respectively. With the exception of **3*a***, which contains one molecule, all the other three contain half molecules in the asymmetric unit. Interestingly no solvent inclusion was found in all four lattices, making the comparison between these structures more realistic. With the exception of **1*a***, the hydrazine moieties (C—N—N—C: 164° in **2*a*** and **3*a*** and 180° in **4*a***) exhibited near co-planar geometry. In **1*a*** the hydrazine moiety resembles that of a simple hydrazine or H_2_O_2_ with C—N—N—C torsion of 115°. The molecules in **1*a*** (along the *c*-axis with a repeat distance of 4.07 Å) and **2*a*** (along the *a*-axis with a repeat distance of 4.73 Å) assemble *via* N—H⋯O hydrogen bonds to form β-sheets containing ten-membered rings. Although both form *β*-sheets the differences in both the structures are apparent given the differences in the geometry of the spacer and molecules: non-coplanar in **1*a*** and nearly planar in **2*a***. In **1*a*** the molecules are aligned in a zigzag manner, while in **2*a*** they are aligned in a plane containing the hydrogen bonds. In **1*a*** the hydrogen bonds [N⋯O, N—H⋯O: 2.823 (3) Å, 173.5°] are more linear than those in **2*a*** (N⋯O, N—H⋯O: 2.817 Å, 147.4°) (Fig. 4 ▸). Given these differences the packing of β-sheets also differ significantly: in **1*a*** the sheets have a parallel packing (C—H⋯π and π⋯π interactions), while in **2*a*** they have herringbone packing (C—H⋯N, C—H⋯π and π⋯π interactions) (Fig. S33).

**Figure 5:**
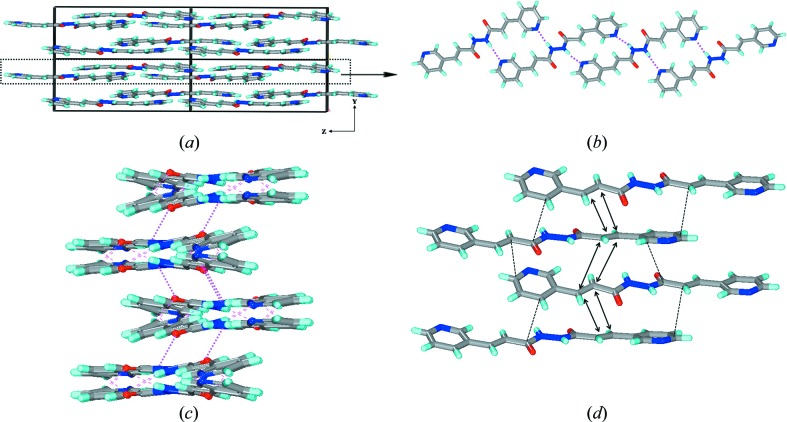
Illustrations for the crystal structure of **3*a***: (*a*) packing diagram; (*b*) one-dimensional chain *via* N—H⋯N hydrogen bonds; (*c*) off-set packing of **3*a***; (*d*) stacking of **3*a*** for single [2 + 2] reaction.

**Figure 6:**
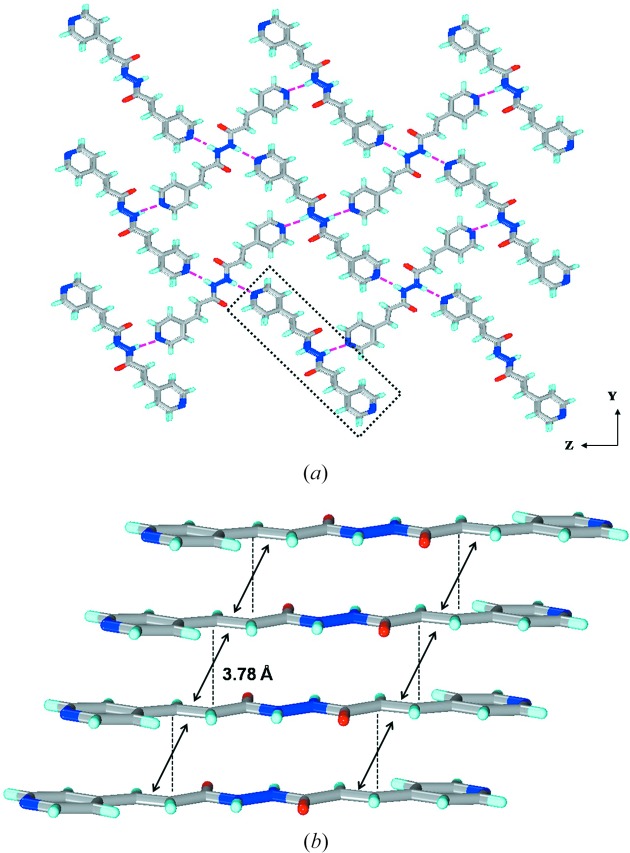
Illustrations for the crystal structure of **4*a***: (*a*) a two-dimensional herringbone layer *via* N—H⋯N hydrogen; (*b*) packing of layers on top of each other to form infinite stacks. Note that the double bonds are aligned within the stacks to undergo double [2 + 2] reaction.

In **3*a*** and **4*a*** the pyridyl groups are found to exhibit interference and form N—H⋯N hydrogen bonds. Although both are nearly planar molecules the hydrogen-bonding patterns and packing are found to differ drastically. In **3*a*** the molecules assemble to form a one-dimensional chain that resembles a β-sheet but *via* N—H⋯N hydrogen bonds and also the molecules are off-set. These one-dimensional chains pack in a parallel fashion which is somewhat similar to that of **1*a*** (Fig. 5 ▸). Whereas in **4a**, the molecules assemble to form a herringbone layer *via* N—H⋯N hydrogen bonds containing rectangular cavities which are filled by the adjacent layers. The layers pack such that the double bonds are aligned for double [2 + 2] reaction (Fig. 6 ▸).

### 3.2. β-sheet, two-dimensional layers and water inclusion with —HN—(CH_2_)_2_—NH— spacer   

The molecules **1*b*** and **2*b*** crystallized in space group *P*2_1_/*c*, while **3*b*** and **4*b*** crystallized in space groups *C*2/*c* and *P*2_1_/*n*, respectively. The asymmetric units of **1*b*** and **4*b*** are constituted by a half unit of the corresponding molecules, while that of **3*b*** is constituted by two half units of **3*b***. The asymmetric unit of **2*b*** contains one full molecule and four H_2_O molecules. The geometries of these molecules were found to be different, although all contain the HN—CH_2_—CH_2_—NH moiety with *anti* geometry (N—C—C—N: 180° in **1*b***, **3*b*** and **4*b*** and 176° in **2*b***). The interplanar angles between the amide planes are 0° in **1*b***, **3*b*** and **4*b***, while it is 61° in **2*b***. Further, the plane of C=C—C=O creates the following angles with the central N—C—C—N plane: 19° in **1*b***, 80° and 19° in **2*b***, 77° and 12° in **3*b*** and 72° in **4*b***.

**Figure 7:**
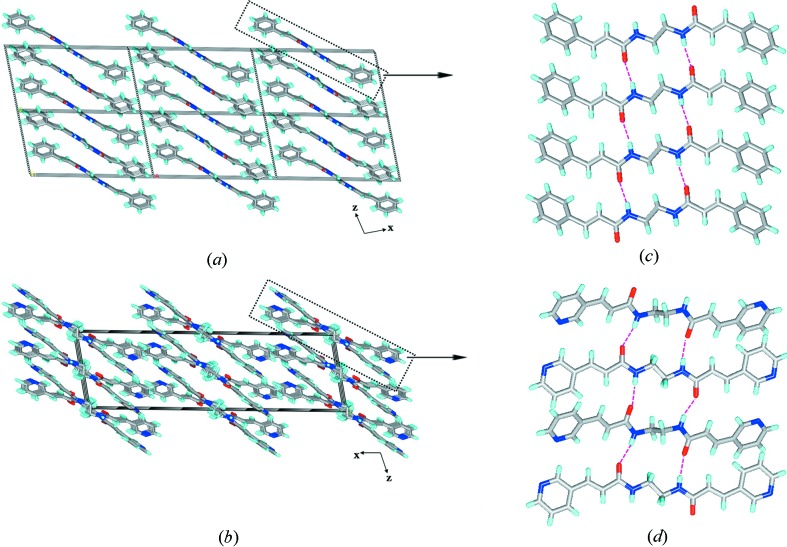
Illustrations for the crystal structures of **1*b*** and **3*b***: packing diagrams for (*a*) **1*b*** and (*b*) **3*b***; β-sheets observed in (*c*) **1*b*** and (*d*) **3*b***. Notice the difference in alignment of molecules within the β-sheet between **1*b*** and **3*b***.

In **1*b*** and **3*b***, the molecules assemble *via* N—H⋯O hydrogen bonds to form the usual β-sheets along the *b*-axis. The molecules within the sheet are in-plane in **1*b*** with a repeat distance of 4.85 Å, while they are not in-plane in **3*b*** and contain a repeat distance of 4.65 Å. The sheets pack in a parallel fashion in the case of **1*b***, while the sheets exhibit some angularity in the packing in **3*b*** (Fig. 7 ▸).

**Figure 8:**
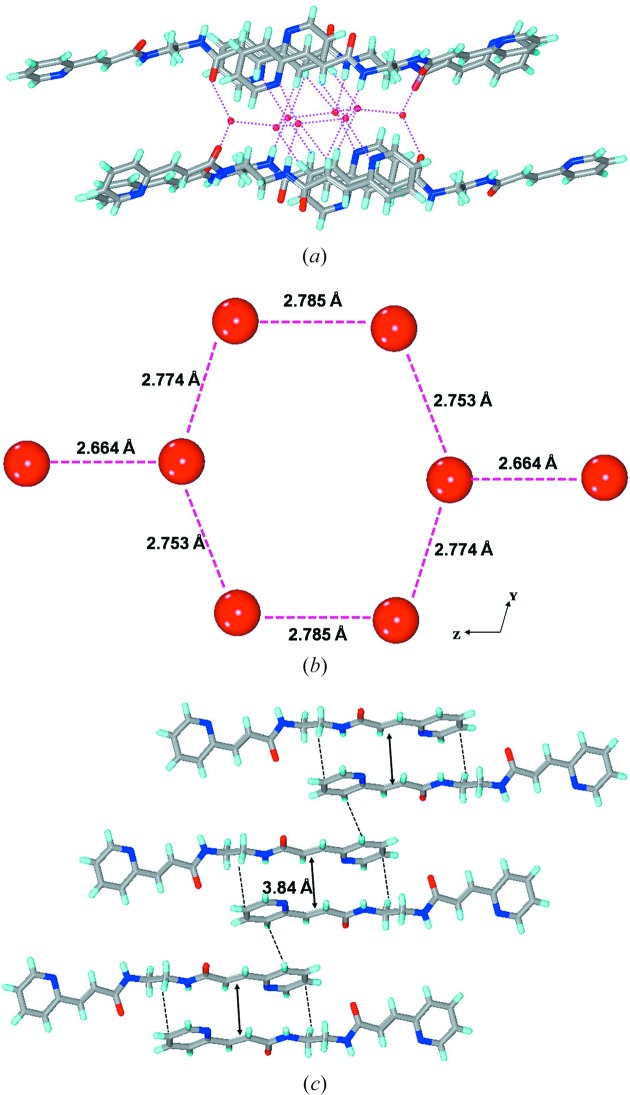
Illustrations for the crystal structure of **2*b***: (*a*) linking of molecules of **2*b*** into the three-dimensional network by the O—H⋯O and N—H⋯O hydrogen bonds with lattice water molecules; (*b*) octameric water cluster *via* O—H⋯O hydrogen bonds; (*c*) alignment of double bonds of **2*b*** for a single [2 + 2] reaction.

The crystal structure of **2*b*** is drastically different from all the structures presented here as the crystal lattice contains four water molecules which govern the overall hydrogen-bonding interactions. No amide-to-amide or amide-to-pyridine hydrogen bonding was found in this structure. The water molecules form an octamer *via* O—H⋯O hydrogen bonds. The octamer contains a flat six-membered ring with the other two water molecules linked at 1,4 positions. These octamers link the molecules of **2*b*** into a three-dimensional network with a plethora of hydrogen-bonding interactions. In the octamer, in terms of hydrogen bonding three types of water molecules exists: (1) four water molecules involved in four hydrogen bonds each, two O—H⋯O with H_2_O, one N—H⋯O*w* with the amide and one O—H⋯N with the pyridine N-atom; these O atoms exhibit near tetrahedral geometry in terms of hydrogen bonding; (2) two involved in exclusively three O*w*—H⋯O*w* hydrogen bonds each with water molecules; (3) two involved in one O*w*—H⋯O*w* and two O*w*—H⋯O=C hydrogen bonds each. The second and third categories exhibit nearly planar geometry in terms of hydrogen bonding. In this three-dimensional network, it was found that the double bonds are aligned for a single [2 + 2] reaction (Fig. 8 ▸).

The crystal structure of **4*b*** bears a close resemblance to that of **4*a*** as it forms a similar herringbone layer *via* N—H⋯N hydrogen bonds. However, here the double bonds are not aligned for the [2 + 2] reaction as the packing of the layers differ due to the presence of the —CH_2_—CH_2_— spacer. Further, this structure is found to be isostructural with that of the amide analogues with 4-pyridyl substitution and the ethyl spacer.

### 3.3. N—H⋯O hydrogen-bonded two-dimensional layers and β-sheet with —HN—(CH_2_)_4_—NH— spacer   

**Figure 9:**
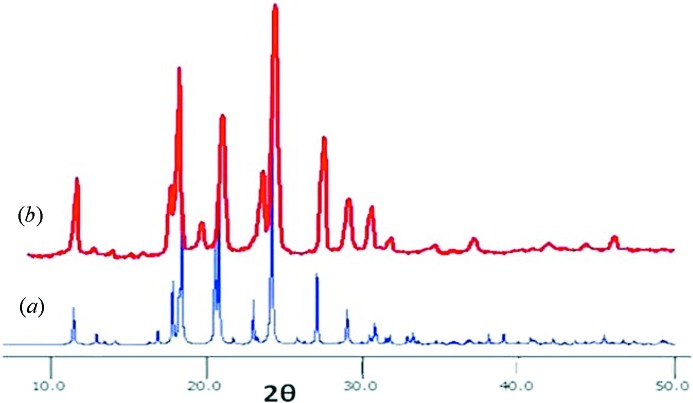
XRPD patterns of (*a*) **1*c*** (calculated); (*b*) **2*c*** (observed); note the matching of patterns.

In this series the single crystals suitable for X-ray diffraction were obtained for **1*c***, **3*c*** and **4*c***. Compound **2*c*** failed to form suitable single crystals despite several trails. The crystal structures and the solid-state reactivates of **1*c*** and **3*c*** were published by us earlier. Molecules **1*c*** and **3*c*** were found to exhibit two-dimensional layers *via* amide-to-amide hydrogen bonds [**1*c***: N⋯O, N—H⋯O: 2.901 (4) Å, 161°] and contain the required double bond alignment for [2 + 2] polymerization as anticipated. In particular, within the layers the double bonds are aligned with a distance (*d*_1_) of 3.812 Å and C=C⋯C=C torsion (τ_2_) of 0°. The comparison of XRPD patterns of **2*c*** with those of **1*c*** indicates that the crystal structures of **2*c*** could be similar to that of **1*c*** (Fig. 9 ▸). Both **1*c*** and **3*c*** contain half molecules in the asymmetric units. The geometries of the molecules of **1*c*** and **3*c*** were found to be somewhat similar to those of the above structures as the planes of C=C—C=O create almost right angles (73.6° in **1*c*** and 74.8° in **3*c***) with that of the spacer —C—C—C—C— plane, and the amide planes are parallel to each other. Further, the central butyl amine moiety is found to have non-planar geometry with the N—C—C—C torsion angles of 59° and 62° in **1*c*** and **3*c***, respectively.

**Figure 10:**
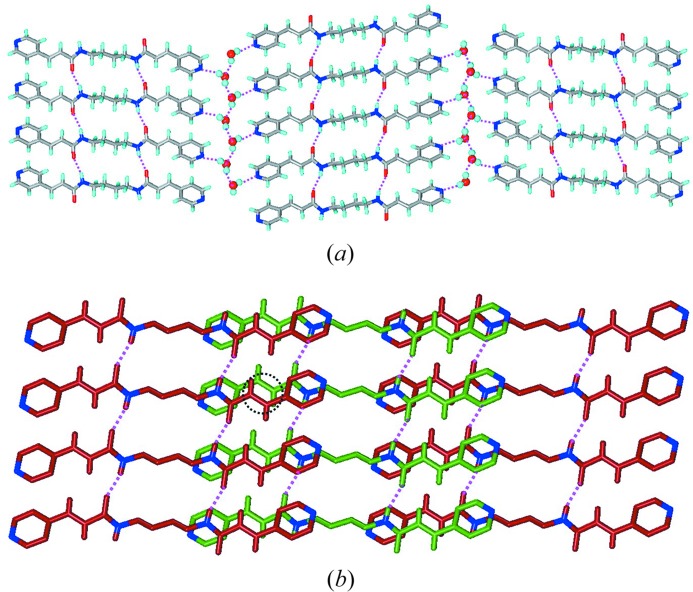
Illustrations for the crystal structure of **4*c***: (*a*) linking of β-sheets *via* H_2_O molecules to form a two-dimensional layer; (*b*) packing of the layers such that the double bonds are aligned for a possible [2 + 2] photopolymerization reaction.

The crystal structure of **4*c*** is found to be totally different from the above structures as it includes water in the crystal lattice. The crystals exhibit space group *P*2_1_/*c* and the asymmetric unit is constituted by half a unit of **4*c*** and one water molecule. The geometry also differs from the above structures as the central plane of the alkyl spacer makes an angle of 64° with that of C=C—C=O. Unlike the above two structures the —N—(CH_2_)_4_—N— fragment exhibits all *anti* geometry with N—C—C—C angles of 180°. The molecules assemble *via* amide-to-amide N—H⋯O hydrogen bonds to form the β-sheet network along the *b* axis with a repeat distance of 4.68 Å. These sheets are further connected *via* O*w*—H⋯N_pyridine_ hydrogen bonds to the chain of water molecules leading to the formation of a two-dimensional layer in which each water has three connectivity (Fig. 10 ▸). These layers stack on each other *via* C—H⋯π and C—H⋯O interactions.

### 3.4. β-sheets with —HN—(CH_2_)_6_—NH— spacer   

**Figure 11:**
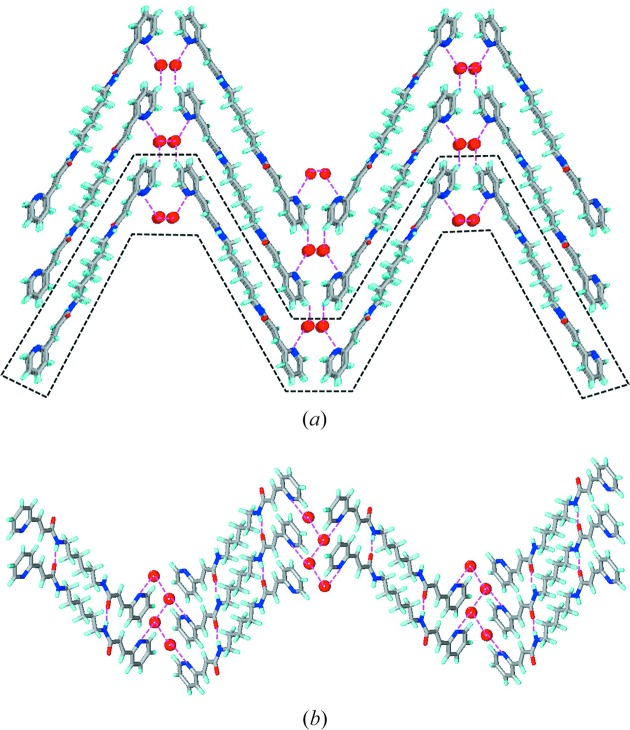
Illustrations for the crystal structure of **2*d***: (*a*) packing diagram, note the linking of β-sheets into the three-dimensional network by water molecules; (*b*) linking of β-sheets of **2*d*** by the zigzag chain of water molecules.

In accordance with our previous studies the introduction of a hexyl spacer resulted in the β-sheet structures in a consistent manner. Molecules **1*d*** and **2*d*** crystallized in *P*2_1_/*c* and **4*d*** crystallized in *P*2_1_/*n*. Single crystals of **3*d*** could not be obtained despite several trails by varying solvents and their combinations. The asymmetric unit of **1*d*** is constituted by one molecule while those of **2*d*** and **4*d*** are constituted by half a unit of the corresponding molecule and one H_2_O molecule. The molecular geometries are somewhat similar in all three cases as the amide planes are in-plane with the plane of hexyl spacer, which exhibits all *anti* geometry. In all three structures the molecules assemble in β-sheets *via* amide-to-amide N—H⋯O hydrogen bonds, along the *b*-axis in **1*d*** and **4*d***, and along the *c*-axis in **2*d*** with the same repeat distance of 4.9 Å. In **2*d*** and **4*d***, neither the water molecules nor the pyridyl groups interfere in amide-to-amide. Rather water molecules form zigzag chains *via* O—H⋯O hydrogen bonds and join the β-sheets in two-dimensional layers *via* O*w*—H⋯N_pyridine_ hydrogen bonds. Interestingly, given the positional differences of pyridines in **2*d*** and **4*d***, the layers have different geometries although they have the same hydrogen-bonding connectivities. In **2*d*** the layers are highly corrugated, while they are planar in **4*d*** (Fig. 11 ▸). In both layers water molecules exhibit 3-connectivity: two O*w*—H⋯O*w* with neighbouring water molecules and O—H⋯N with pyridine moieties. We note here that the crystal structure of **4*d*** was found to be isostructural with that of **4*c***. The layers pack on each other with an interlayer separation of 4 Å in **2*d*** and **4*d***, whereas in **1*d*** the β-sheets pack in a parallel fashion. The XRPD of **3*d*** was found to be similar to that of **1*d***, indicating it may also have similar β-sheet formation (Fig. S30).

### 3.5. Molecular structure *versus* hydrogen-bonding networks   

In previous studies on amides and reverse amides it was found that the geometry of the molecule and position of the pyridine groups play a key role in the competition of acceptors C=O of amide and the N atom of pyridine to form hydrogen bonds with the N—H group of amides. In particular, the inter-planar angle (θ) between the amide group and the terminal aromatic ring tailors the resultant hydrogen bonds. The θ-value of less than 20° results in the formation of N—H⋯N hydrogen bonds, while above 20° results in the formation of N—H⋯O hydrogen bonds. This phenomenon was also explored using a larger set of compounds from the CSD. In the present series it was found that this hypothesis holds good with the exception of three compounds, namely **1*a***, **1*c*** and **3*c***, which form amide-to-amide hydrogen bonds despite having a θ-value below 20°, *i.e.* 8.45°, 12.83° and 8.42°, respectively (Table S2).

The probable reasons could be due to their unusual molecular geometries which differ totally from the rest of the structures presented here as they deviate heavily from linearity. Further, it was found earlier that the phenyl and the 3-pyridyl substituted derivatives do not form hydrates, while the 4-pyridyl derivatives do exhibit such a tendency. Similarly, out of four 4-pyridyl derivatives studied here, two form hydrates. Interestingly, the present studies show that the 2-pyridyl groups also have a similar tendency to form such hydrates as two out of three form hydrates.

### 3.6. Trends in the melting points of the homologues series   

**Figure 12:**
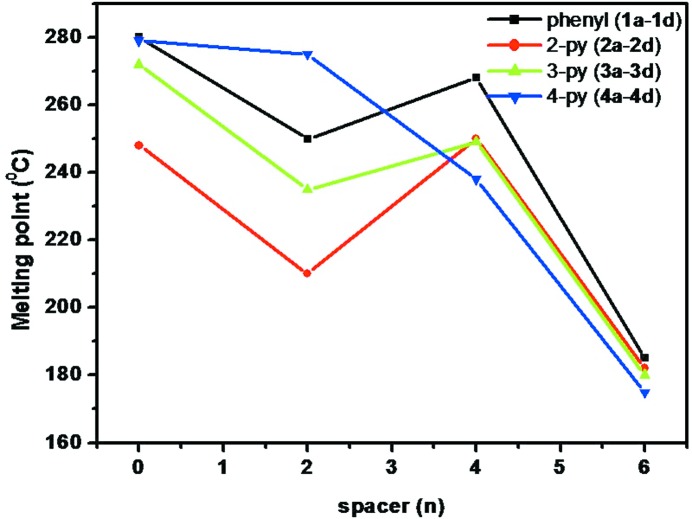
Trends observed in the melting points of the homologous series.

We are in agreement with the general conception that the decrease in melting points was observed with an increase in the number of CH_2_ groups, with the caveat that the derivatives with a butyl spacer (**1*c***, **2*c***, **3*c*** and **4*c***) deviate from such trends (Hall & Reid, 1943 ▸; Thalladi, Boese & Weiss, 2000 ▸; Thalladi, Nüsse & Boese, 2000 ▸). The butyl derivatives **1*c***, **2*c*** and **3*c*** are found to exhibit higher melting points than the corresponding ethyl (**1*b***, **2*b***, **3*b***) and hexyl derivatives (**1*d***, **2*d***, **3*d*** and **4*d***). A further decrease in the melting point was observed for hydrates compared with their respective analogues. Phenyl and 4-pyrydyl derivatives have been shown to have higher melting points than the 3-pyridyl and 2-pyridyl derivatives. A similar tendency was also observed in the melting points of amides and reverse amides (Mukherjee & Biradha, 2011*a* ▸). No correlation of melting points was observed with either the dimensionality of the network or nature of the hydrogen bonds (N—H⋯O *versus* N—H⋯N) (Fig. 12 ▸). Further, in a given series a linear increase in the densities of the derivatives was observed with an increase in the number of —(CH_2_)— groups with the exception of **1*a***. Interestingly, the densities of 4-pyridyl derivatives are found to be higher than those of the other three homologues. In contrast, the homologous series containing a phenyl substituent was found to exhibit lower densities than the other three series (Fig. S38).

### 3.7. Solid-state reactivities of **1*c***, **2*b***, **2*c***, **3*a***, **4*a***, **3*c*** and **4*c***   

**Figure 13:**
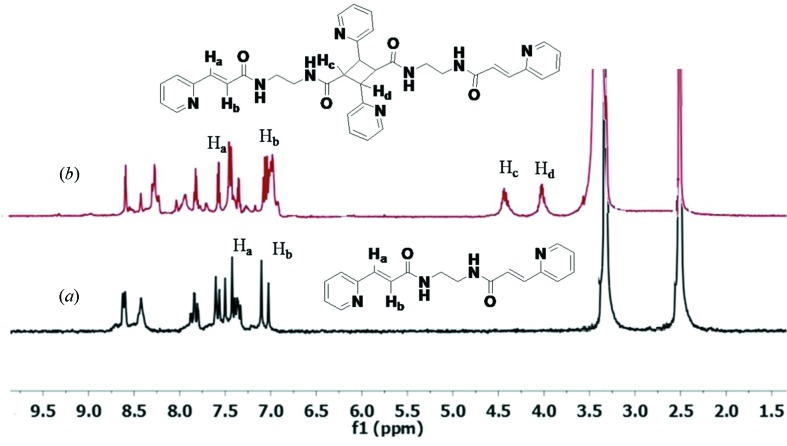
^1^H NMR in DMSO-d^6^ (*a*) of **2*b***, the peaks at 7.45 and 7.04 p.p.m. represent olefin protons; (*b*) of the single dimer of **2*b***, the presence of cyclobutane protons at 4.44 and 4.03 p.p.m. with unreacted olefin doublet (H_a_ and H_b_) confirms the single [2 + 2] product.

The crystal structure analysis of **2*b*** reveals that the double bonds are aligned for a single [2 + 2] reaction with a C⋯C distance (*d*_1_) between the double-bonded C atoms of 3.84 Å and C=C⋯C=C torsion of (τ_2_) 0°. The ^1^H NMR spectra of irradiated **2*b*** in DMSO-d^6^ shows the appearance of cyclobutane protons at 4.44 and 4.03 p.p.m. with the presence of olefinic protons. From these observations it can be concluded that **2*b*** was converted to a single dimer product in 59% yield after 72 h of irradiation in sunlight (Fig. 13 ▸).

As it was described earlier, the materials **1*c***, **2*c***, **3*c*** and **4*c*** have a required alignment for solid-state [2 + 2] polymerization reactions. The compounds **1*c*** and **3*c*** were shown by us earlier to undergo a polymerization reaction in a SCSC manner to yield crystalline covalent polymers. Although single crystals of **2*c*** were not obtained, the comparison of XRPD patterns of **2*c*** revealed that it also contains similar packing with two-dimensional (4,4)-layers as in **1*c*** and **3*c***. Therefore, a polycrystalline material of **2*c*** was irradiated in sunlight for 96 h. The irradiated product was found to be insoluble in common organic solvents similar to those of **1*c*** and **3*c***. However, it was found to be soluble in DMSO or aqueous solution with a drop of HCl, HNO_3_ or H_2_SO_4_.

**Figure 14:**
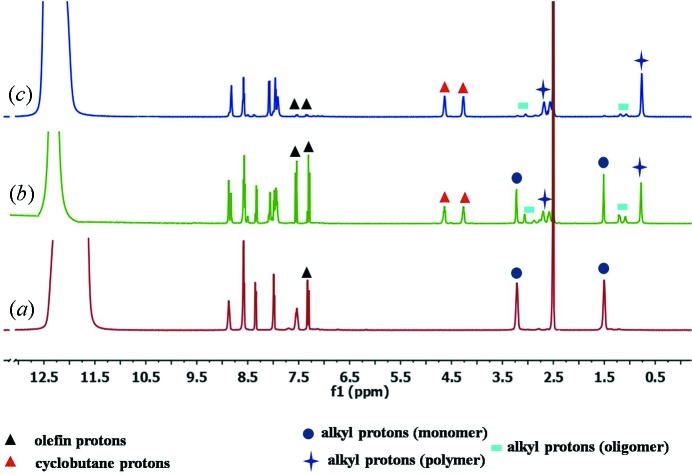
^1^H-NMR spectra recorded at various stages of irradiation of compound **2*c***: (*a*) before irradiation, (*b*) after irradiation (monomer, oligomers and polymer) and (*c*) after separation of polymer from monomer and oligomers.

The ^1^H NMR spectra of an irradiated sample of **2*c*** in DMSO-d^6^ and one drop of H_2_SO_4_ revealed that the reaction proceeds through the formation of oligomers as the resultant spectra contains some new peaks in addition to the peaks of the monomer and polymer. In the spectra the cyclobutane peaks of a polymer appeared at 4.27 and 4.64 p.p.m. and also the *n*-butyl protons of the polymer were found to exhibit an up-field shift from monomer to polymer, *i.e.* 3.22 to 2.56 p.p.m. and 1.51 to 0.77 p.p.m. For oligomers, the cyclobutane peaks appeared at the same p.p.m. as the polymer, however, the corresponding *n*-butyl peaks appeared at 3.05, 1.22 and 1.08 p.p.m.. From ^1^H NMR, the yield of the reaction including oligomers was found to be 45%. The result of the polymerization reaction observed here is in line with that of **3*c*** albeit the yield of the polymer is not 100% in the present case. The unreacted **2*c*** and oligomers were removed by repeatedly washing the irradiated material of **2*c*** with hot methanol. The ^1^H NMR of the separated polymer in DMSO-d^6^ with one drop of H_2_SO_4_ reveals the presence of cyclobutane protons at 4.27 and 4.64 p.p.m. with *n*-butyl protons at 2.57 and 0.77 p.p.m. and the absence of olefin protons (Fig. 14 ▸). However, unlike the polymer of **3*c***, the polymeric material of **2*c*** does not result in the formation of plastic films.

**Figure 15:**
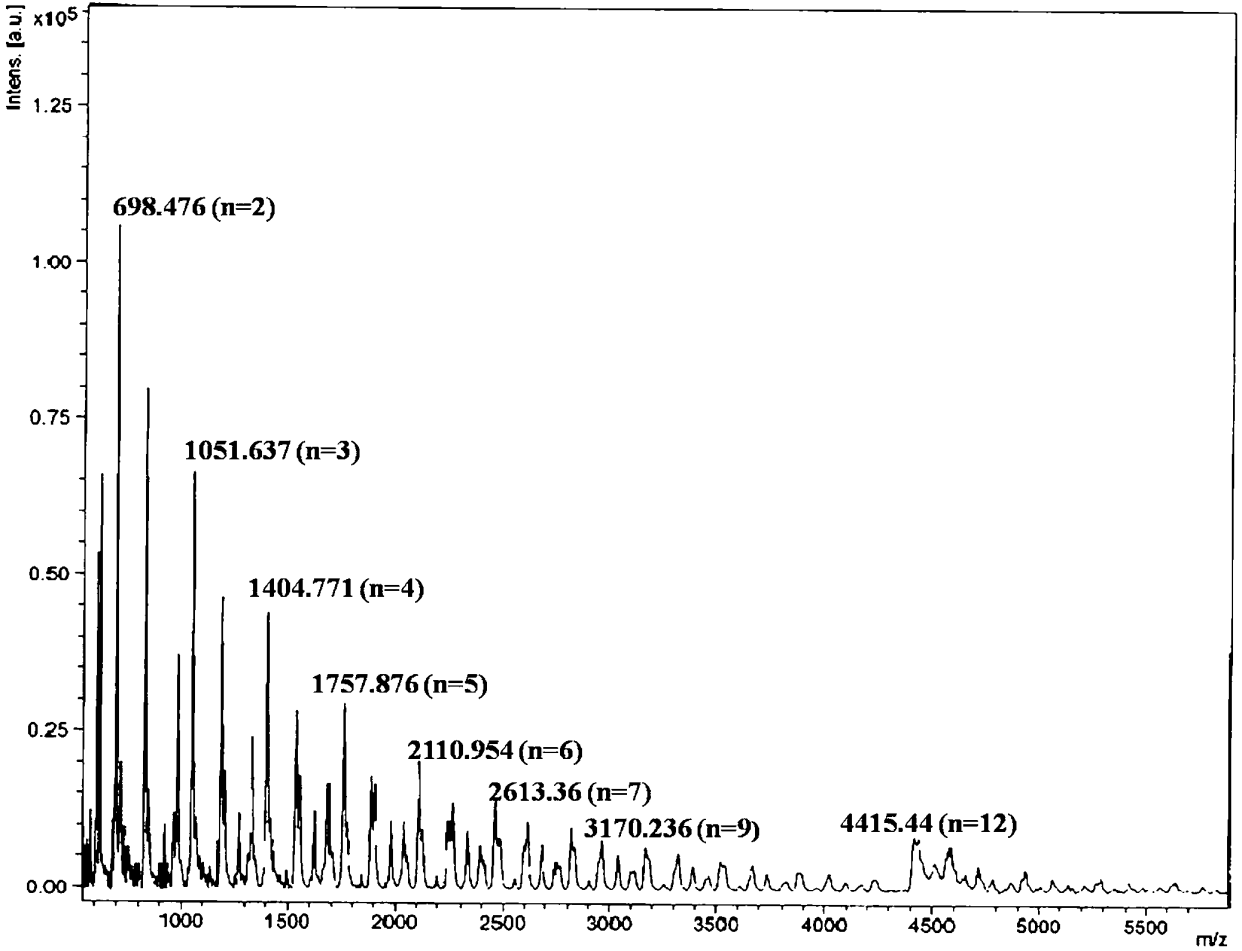
MALDI-TOF mass data for **2*c*** (irradiated).

The molecular weight of the polymer (**2*c***) was determined using MALDI-TOF (matrix-assisted laser desorption/ionization time-of-flight) with 2,5-dihydroxy benzoic acid (DHB) as a matrix in solution. The highest molecular ion peak was observed at 4415 (*m*/*z*), which corresponds to 12-*mer* of **2*c*** (Fig. 15 ▸).

Although **4*c*** forms β-sheet type N—H⋯O hydrogen bonds, the double bonds of **4*c*** are aligned with the other layer and fulfil [2 + 2] photopolymerization criteria with appropriate geometrical parameters: *d*_1_ = 4.18 Å and τ_2_ = 0°. However, **4*c*** was found to be photostable even after prolonged irradiation. The probable reason could be loss of the lattice water which triggers the structural transformation to an unreactive form. Indeed, it was found that the XRPD pattern of **4*c*** which is recorded at room temperature does not match the calculated XRPD pattern of **4*c*** indicating the loss of water. The single-crystal data for this material was collected at low temperature.

Similarly, compounds **3*a*** and **4*a*** were found to be photostable despite the possibility of single and double [2 + 2] reactions, respectively. The *d*_1_ and τ_2_ values for **3*a*** and **4*a*** are 3.909 Å and 0.74°, and 3.779 Å and 0°, respectively. The probable reason could be that in **3*a*** the double bonds exhibit the higher displacement value of 1.9 Å, whereas in **4*a*** the existence of infinite stacks rather than discrete dimers might have prevented the reaction (Gnanaguru *et al.*, 1985 ▸; Murthy *et al.*, 1987 ▸; Nagarathinam *et al.*, 2008 ▸; Yang *et al.*, 2009 ▸).

## 4. Conclusions   

The homologous series exhibit variation in their crystal structures depending upon the spacers and end groups such as phenyl, 2-pyridyl, 3-pyridyl or 4-pyridyl. Unlike in previously studied series, in the current one the hydrazine spacer and 2-pyridyl derivatives were included for the first time. Some of the structural aspects observed here have direct correlations with those of amide/reverse amide homologues. For example, amide derivatives with a butyl spacer have previously been shown to form two-dimensional layers *via* N—H⋯O hydrogen bonds for both 3-pyridyl and 4-pyridyl derivatives (Sarkar & Biradha, 2006 ▸). Similarly, in the current study the phenyl, 2-pyridyl and 3-pyridyl derivatives containing a butyl spacer form such a two-dimensional N—H⋯O hydrogen-bonded layer. However, the 4-pyridyl derivative deviates as it forms β-sheets which are linked further by water molecules. Further, all the derivatives containing a hexyl spacer exhibited β-sheets irrespective of the end attachments, which is in agreement with the amide derivatives containing a hexyl spacer and 3-pyridyl or 4-pyridyl attachments.

## Supplementary Material

Crystal structure: contains datablock(s) 1a, 1b, 1d, 2a, 2b, 2d, 3a, 3b, 4b, 4c, 4d. DOI: 10.1107/S2052252515009987/yc5005sup1.cif

Structure factors: contains datablock(s) kb_mg_080. DOI: 10.1107/S2052252515009987/yc50051asup2.hkl

Structure factors: contains datablock(s) kb_mg_2. DOI: 10.1107/S2052252515009987/yc50051bsup3.hkl

Structure factors: contains datablock(s) kbmg109. DOI: 10.1107/S2052252515009987/yc50051dsup4.hkl

Structure factors: contains datablock(s) kbmg181. DOI: 10.1107/S2052252515009987/yc50052asup5.hkl

Structure factors: contains datablock(s) kbmg146. DOI: 10.1107/S2052252515009987/yc50052bsup6.hkl

Structure factors: contains datablock(s) kbmg154. DOI: 10.1107/S2052252515009987/yc50052dsup7.hkl

Structure factors: contains datablock(s) kb_rs_501. DOI: 10.1107/S2052252515009987/yc50053asup8.hkl

Structure factors: contains datablock(s) kbmg011. DOI: 10.1107/S2052252515009987/yc50053bsup9.hkl

Structure factors: contains datablock(s) kbgt001. DOI: 10.1107/S2052252515009987/yc50054bsup10.hkl

Structure factors: contains datablock(s) kbmg176. DOI: 10.1107/S2052252515009987/yc50054csup11.hkl

Structure factors: contains datablock(s) kbmg112. DOI: 10.1107/S2052252515009987/yc50054dsup12.hkl

CCDC references: 1055532, 1055533, 1055534, 1055535, 1055536, 1055537, 1055538, 1055539, 1055540, 1055541, 1055542

## Figures and Tables

**Table:** 

	**1*a***	**1*b***	**1*d***	**2*a***	**2*b***	**2*d***
Chemical formula	C_18_H_16_N_2_O_2_	C_20_H_20_N_2_O_2_	C_24_H_28_N_2_O_2_	C_16_ H_14_N_4_O_2_	C_18_H_18_N_4_O_6_	C_22_H_26_N_4_O_4_
*M*_r_	292.33	320.38	376.48	294.31	394.43	414.50
*T* (K)	293	293	293	100	293	293
Crystal system	Monoclinic	Monoclinic	Monoclinic	Monoclinic	Monoclinic	Monoclinic
Space group	*C*2/*c*	*P*2_1_/*c*	*P*2_1_/*c*	*P*2_1_/*n*	*P*2_1_/*c*	*P*2_1_/*c*
*a* ()	18.451(9)	17.899(3)	20.243(5)	4.7315(10)	9.3530(6)	7.6364(14)
*b* ()	10.540(5)	4.8544(9)	4.9756(14)	9.213(2)	10.9015(7)	30.058(5)
*c* ()	8.157(4)	9.5259(16)	20.839(6)	16.390(4)	19.9985(14)	4.8885(9)
()	90.00	90.00	90.00	90.00	90.00	90.00
()	98.824(12)	101.847(4)	99.148(8)	97.020(6)	94.547(2)	95.523(6)
()	90.00	90.00	90.00	90.00	90.00	90.00
*V* (^3^)	1567.6(13)	810.1(2)	2072.2(10)	709.1(3)	2032.7(2)	1116.9(3)
*Z*	4	2	4	2	4	2
*D*_*x*_ (mgm^3^)	1.239	1.313	1.207	1.378	1.289	1.233
*R*_1_ [*I* > 2(*I*))]	0.0549	0.00373	0.0785	0.0401	0.0638	0.0714
*wR*_2_ (on *F* ^2^, all data)	0.1930	0.1071	0.2013	0.1319	0.2030	0.1999

**Table:** 

	**3*a***	**3*b***	**4*b***	**4*c***	**4*d***
Chemical formula	C_16_H_14_N_4_O_2_	C_18_H_18_N_4_O_2_	C_18_H_18_N_4_O_2_	C_20_H_26_N_4_O_4_	C_22_H_30_N_4_O_4_
*M*_r_	294.31	322.36	322.36	386.45	414.50
*T* (K)	293	293	293	100	293
Crystal system	Monoclinic	Monoclinic	Monoclinic	Monoclinic	Monoclinic
Space group	*P*2_1_/*c*	*C*2/*c*	*P*2_1_/*n*	*P*2_1_/*c*	*P*2_1_/*n*
*a* ()	10.403(6)	34.566(5)	4.7967(15)	12.78(2)	7.3720(7)
*b* ()	7.160(4)	9.3338(13)	11.547(4)	4.681(9)	4.9035(4)
*c* ()	19.202(11)	10.4191(15)	14.726(5)	16.21(3)	31.586(3)
()	90.00	90.00	90.00	90.00	90.00
()	101.156(17)	100.401(4)	96.451(10)	93.42(3)	90.197(3)
()	90.00	90.00	90.00	90.00	90.00
*V* (^3^)	1403.1(14)	3306.3(8)	810.5(4)	968(3)	1141.78(18)
*Z*	4	8	2	2	2
*D_x_* (mgm^3^)	1.393	1.295	1.321	1.326	1.206
*R*_1_ [*I* > 2(*I*)]	0.0430	0.0599	0.0646	0.0853	0.0599
*wR*_2_ (on *F* ^2^, all data)	0.0801	0.2079	0.1403	0.2241	0.2079

**Table 2:** Geometrical parameters (, ) of hydrogen bonds

	Type	H*A* ()	*D* *A* ()	*D*H*A* ()
**1*a***	NHO	1.97	2.823(3)	173
	CHO	2.51	2.843(3)	101
**1*b***	NHO	2.04	2.889(2)	171
**1*d***	NHO	2.13	2.974(4)	166
	NHO	2.09	2.939(4)	170
	CHO	2.53	2.858(5)	101
**2*a***	NHO	2.06	2.817	147
	CHN	2.58	3.442(6)	155
	CHO	2.58	3.400(3)	148
	CHO	2.54	2.847(5)	100
**2*b***	NHO	2.20	3.056(3)	174
	NHO	2.23	3.037(3)	156
	CHO	2.47	2.809(3)	101
	CHO	2.49	3.381(3)	160
	CHO	2.56	2.875(3)	100
	CHO	2.43	2.767(3)	100
**2*d***	NHO	2.11	2.943(4)	162
	CHO	2.60	3.443(6)	152
	CHO	2.51	2.838(5)	101
**3*a***	NHO	2.20	3.032(3)	163
	NHO	2.32	3.165(3)	169
	CHO	2.46	3.144(3)	131
	CHO	2.50	2.839(3)	101
	CHO	2.55	3.222(3)	130
	CHO	2.50	3.198(3)	132
	CHO	2.50	2.841(3)	102
**3*b***	NHO	2.06	2.910(3)	167
	NHO	1.97	2.809(3)	166
	CHO	2.49	2.828(3)	101
	CHO	2.52	2.848(3)	101
**4*b***	NHN	2.15	2.980(5)	161
	CHO	2.48	3.219(6)	137
	CHO	2.54	2.861(5)	100
	CHO	2.45	2.825(5)	103
**4*c***	OHO	1.83(5)	2.763(7)	153(3)
	OHN	2.06(4)	2.805(7)	169(5)
	NHO	1.97	2.803(7)	164
	CHO	2.42	3.112(8)	131
	CHO	2.52	3.463(8)	165
	CHO	2.45	2.822(7)	103
**4*d***	NHO	2.07	2.921(2)	168
	OHN	2.02(3)	2.891(4)	169.3(19)
	CHO	2.53	2.849(3)	100
